# National Institutes of Health Clinical Research Funding and All-Cause In-Hospital Traumatic Brain Injury-Related Mortality

**DOI:** 10.7759/cureus.27228

**Published:** 2022-07-25

**Authors:** Anwar Alinani, Brianna Mills, Emma Gause, Monica S Vavilala, Abhijit V Lele

**Affiliations:** 1 Anesthesiology and Pain Medicine, Harborview Medical Center, Seattle, USA; 2 Anesthesiology and Pain Medicine, Harborview Injury Prevention and Research Center, Seattle, USA; 3 Anesthesiology and Pain Medicine, Harborview Injury Prevcention and Research Center, Seattle, USA; 4 Neurocritical Care/Anesthesiology, Harborview Medical Center, Seattle, USA

**Keywords:** outcome research, traumatic brain injury, severe, mortality, funding, in-hospital

## Abstract

Introduction

Higher federal research funding levels may improve patient outcomes. We examined this relationship between traumatic brain injury (TBI) funding and all-cause in-hospital TBI-related mortality.

Methods

Using an ecological series analysis, we examined the linear trend in both clinical TBI research funding in year 2000 United States dollars ($) (National Institutes of Health [NIH] RePORTER) and in-hospital isolated TBI mortality among patients aged 15 and older (National Trauma Data Bank [NTDB], TBI-related ICD-9 or ICD-10 code, abbreviated head injury score >2 and body region score <2 with ICU admission) between 2007-2015 with data from centers contributing all years of data for the study period. Linear regression was used to assess the relationship between mortality rate and total funding, lagged one to three years, both overall and within ten-year age groups.

Results

The mean annual NIH-TBI research funding was $64.36 million (lowest: 2008; $48.79 million, highest: 2015; $71.42 million). 192,597 encounters of patients 15 years and older, predominantly male (67.5%) and with polytrauma (59.9%), were included. There was no statistically significant reduction in in-hospital TBI-related mortality (14.15% in 2007 to 13.36% in 2015) for the cohort overall, but the mortality rate decreased for patients over 55 years. The greatest mortality reduction occurred in patients 85 years and older (-62.35, 95% CI -92.45-32.25), followed by patients 75-84 years (-44.41, 95% CI -61.72, -27.09), patients 65-74 years (-47.60, 95% CI -67.39, -27.81), and patients 55-64 years of age (-15.15, 95% CI -27.59, -2.72). During the study period, annual NIH funding for TBI varied from the lowest level of $48.79 million (in 2007) to the highest level of $77.34.43 million (in 2005). There was no association between funding in the previous three years and the in-hospital TBI-related mortality rate.

Conclusion

This study found a variable pattern in NIH funding for clinical TBI research and a contemporaneous reduction in moderate-severe TBI-related deaths only for those aged 55 years and older, but no association between funding and mortality.

## Introduction

Traumatic brain injury (TBI) is a major cause of global death and disability, and in the U.S. alone, TBI contributes to about 30% of all injury deaths [1]. In 2014, TBI accounted for 288,000 hospitalizations and 56,800 deaths [2]. According to the Centers for Disease Control and Prevention’s (CDC) National Center for Health Statistics (NCHS), the National Hospital Ambulatory Medical Care Survey (NHAMCS), and the National Hospital Discharge Survey (NHDS), the overall (composite of all TBI severity) case fatality rate of TBI was 30.5% in 2010 [3], with significant mortality (Abbreviated Injury Score 4-6 [4], hazard ratio 1.24, p<.001) attributed to severe TBI [5]. The 2014 combined financial burden of TBI-related emergency department visits, hospitalizations, and deaths was $2.87 million in 2013. The direct cost of TBI was estimated to be $13.1 billion, while an additional $64.7 billion was lost through missed work and lost productivity [6]. Despite research funding to improve TBI care and outcomes, TBI mortality rates remain high.

The National Institutes of Health (NIH), under the oversight of the Congress, uses the burden of disease as the most important factor in making decisions regarding funding allocations. Other possible funding considerations include recent changes in the burden of disease, the potential for a scientific breakthrough and advocacy by disease-focused organizations. NIH funding has increased more slowly than growth in the gross domestic product in recent years. Hence, the rationale for NIH allocations for specific diseases is critical [7].

The United States has a large TBI research portfolio, but the survival impact of federally funded research on patient outcomes is not well understood. One study analyzed the allocation of global investments from public and philanthropic funders for blast injury-related research and blast TBI between 2000 and 2019. While the authors concluded that blast TBI research received 42.6% ($384..3 million) of all blast-injury-related research [8], the study did not examine concurrent trends in TBI mortality. Thus, in our study, we examined recent trends in the National Institutes of Health, NIH-funded clinical TBI research and concurrent TBI-related in-hospital mortality. We hypothesized that NIH funding for TBI increased over time and that increases in funding would have been associated with reduced in-hospital TBI mortality.

## Materials and methods

Human subjects approval

Since this work was considered non-human subjects research, this work was deemed to be exempt by the University of Washington Institutional Review Board [9]. 

Study design

This was a retrospective, ecological time-series analysis examining the relationship between all-cause in-hospital mortality among isolated TBI patients and NIH funding within the prior three years in the U.S., as well as their individual trends over time.

Exposure

The main exposure of interest was NIH funding for TBI research, adjusted to year 2000 United States dollars ($), data extracted from NIH. The NIH RePORTER [10] was queried on May 28, 2018, for projects and funding levels related to clinical TBI research between the fiscal years 2000 and 2015 using search terms: traumatic brain injury. We excluded research conducted on children and animals. The funding sources are listed in Table 1. 

**Table 1:** NIH RePORTER search funding sources NIH: National Institutes of Health, RePORTER: NIH-search box

Abbreviation	Funding Agency
FIC	Fogarty International Center
NICHD	Eunice Kennedy Shriver National Institute of Child Health and Human Development
NCI	Nonviolent Crisis Intervention
NCATS	National Center for Advancing Translational Sciences
NCCIH	National Center for Complementary and Integrative Health
CAM	Complementary and Alternative Medicine
NCRR	National Center for Research Resources
NEI	National Eye Institute
NHLBI	The National Heart, Lung, and Blood Institute
NHGRI	National Human Genome Research Institute
NIAID	National Institute of Allergy and Infectious Diseases
NIAMS	National Institute of Arthritis and Musculoskeletal and Skin Diseases
NIBIB	National Institute of Biomedical Imaging and Bioengineering
NIDCR	National Institute of Dental and Craniofacial Research
NIDDK	National Institute of Diabetes and Digestive and Kidney Diseases
NIEHS	National Institute of Environmental Health Sciences
NIGMS	National Institute of General Medical Sciences
NIMH	National Institute of Mental Health
NINDS	National Institute of Neurological Disorders and Stroke
NINR	National Institute of Nursing Research
NIA	National Institute on Aging
NIAAA	National Institute on Alcohol Abuse and Alcoholism
NIDCD	National Institute on Deafness and Other Communication Disorders
NIDA	National Institute on Drug Abuse
NIMHD	National Institute on Minority Health and Health Disparities
NIMHD	National Institute on Minority Health and Health Disparities
NLM	National Library of Medicine
CIT	Center for Information Technology
CSR	Center for Scientific Review
CLC	Clinical Center
OD	Office of the Director, NIH
WH	Women's Health Initiative

Outcome

The primary outcome of interest was in-hospital mortality among patients with moderate-severe isolated TBI injuries as captured in the National Trauma Data Bank (NTDB) discharge disposition variable. Patients who died in the hospital or who were transferred to hospice were included in the mortality rate. Mortality was measured as the rate of in-hospital deaths per 10,000 isolated TBI hospitalizations. Data from patients hospitalized with TBI were extracted from the NTDB from 2007 to 2015. Since calculated abbreviated injury scores were not included in the NTDB database after 2015, we restricted our study period to 2007-2015, when data were available. Participating trauma centers from across the United States voluntarily submit their trauma data to the NTDB to improve trauma research. Isolated TBI encounters within NTDB were defined using the following criteria: TBI-related ICD-9 or ICD-10 code (Table 2), head abbreviated injury score (AIS)>2, no other body region AIS>2, and ICU admission during the encounter. Patients aged 15 years or older were included in the study. For consistency throughout the study period, only data from trauma centers that reported to NTDB in all nine years were included in the study.

**Table 2:** ICD-9 and ICD-10 codes used to classify TBI injuries ICD: International Classification of Diseases, TBI: traumatic brain injury

Code Type	ICD Code	Definition
ICD-10	S01	Open wound of the head
ICD-10	S02.0, S02.1, S02.3, S02.8–S02.9	Fracture of the skull and facial bones
ICD-10	S04.0	Injury to optic nerve and pathways
ICD-10	S06	Intracranial injury
ICD-10	S07	Crushing injury of head
ICD-10	S09.8–S09.9	Other unspecified injuries of the head
ICD-9	850	Concussion
ICD-9	851	Cerebral laceration and contusion
ICD-9	852	Subarachnoid subdural and extradural hemorrhage following injury
ICD-9	853	Other and unspecified intracranial hemorrhage following injury
ICD-9	854	Intracranial injury of other and unspecified nature
ICD-9	800	Fracture of vault of the skull (excluding 800.5: Open fracture of vault of the skull without mention of intracranial injury)
ICD-9	801	Fracture of base of the skull (excluding 801.5: Open fracture of base of skull without mention of intracranial injury)
ICD-9	803	Other and unqualified skull fractures (excluding 803.5: Other open skull fracture without mention of intracranial injury)
ICD-9	804	Multiple fractures involving skull or face with other bones (excluding 804.5: Open fractures involving skull or face with other bones without mention of intracranial injury)

Statistical analysis

These analyses consisted of three parts. First, NIH funding for TBI-related research within the last 12 years was assessed descriptively from 2004 through 2015. The same was done for in-hospital mortality during the study years of 2007 through 2015 overall and within ten-year age groups. Results were graphed to visually inspect the pattern of funding and mortality rates over time. The proportions of encounters that resulted in an in-hospital death or transferred to hospice, as well as encounters that resulted in a good outcome, were calculated. A “good” outcome was defined as encounters with a discharge disposition that did not result in death or substantial further care, either intermediate or long-term.

A series of bivariate linear regression models with time as the independent variable were then fit to the mortality data, both overall and within ten-year age groups, to evaluate the linear trend in the mortality rate per 10,000 hospitalized moderate to severe, isolated TBI injuries over the study period from 2007 through 2015. The exposure was centered so that the intercept represented the chronological year 2007.

The data were then stratified into eight subsets by ten-year age groups from 15-25-year-old patients up to 85+-year-old patients. The same bivariate linear regression models were repeated within each age group to identify whether trends in mortality differed by patient age. Lastly, the association between TBI-related mortality rates and TBI-related funding from the prior one to three years was assessed using a series of time series linear regression models. The first model was fit to the full dataset where the overall TBI-related mortality rate in any given year was included as the outcome, and TBI-related funding, which lagged one to three years, was incorporated as the exposure. This model was then applied to the data stratified by age group to assess whether the association between previous years’ funding and mortality differed by patient age. A global F test was used to determine the statistical significance of all lagged funding variables in each model. All analyses were conducted in Stata Version 13 [11].

## Results

Cohort characteristics

The majority of patients were male (67.5%), 39.8% were transferred from referral facility, and 59.9% had polytrauma, meaning they had a documented AIS score for another body region other than the head that was less than or equal to 2. Patients had a median intensive care unit (ICU) length of stay of 4.4, interquartile range (IQR) of 1-5 days and a median hospital length of stay of 8.3, IQR of 3-10 days. Overall, 169,353 (59.4%) had a good discharge outcome, and 39,506 (13.8%) patients either died or were discharged to hospice. 

**Figure 1:**
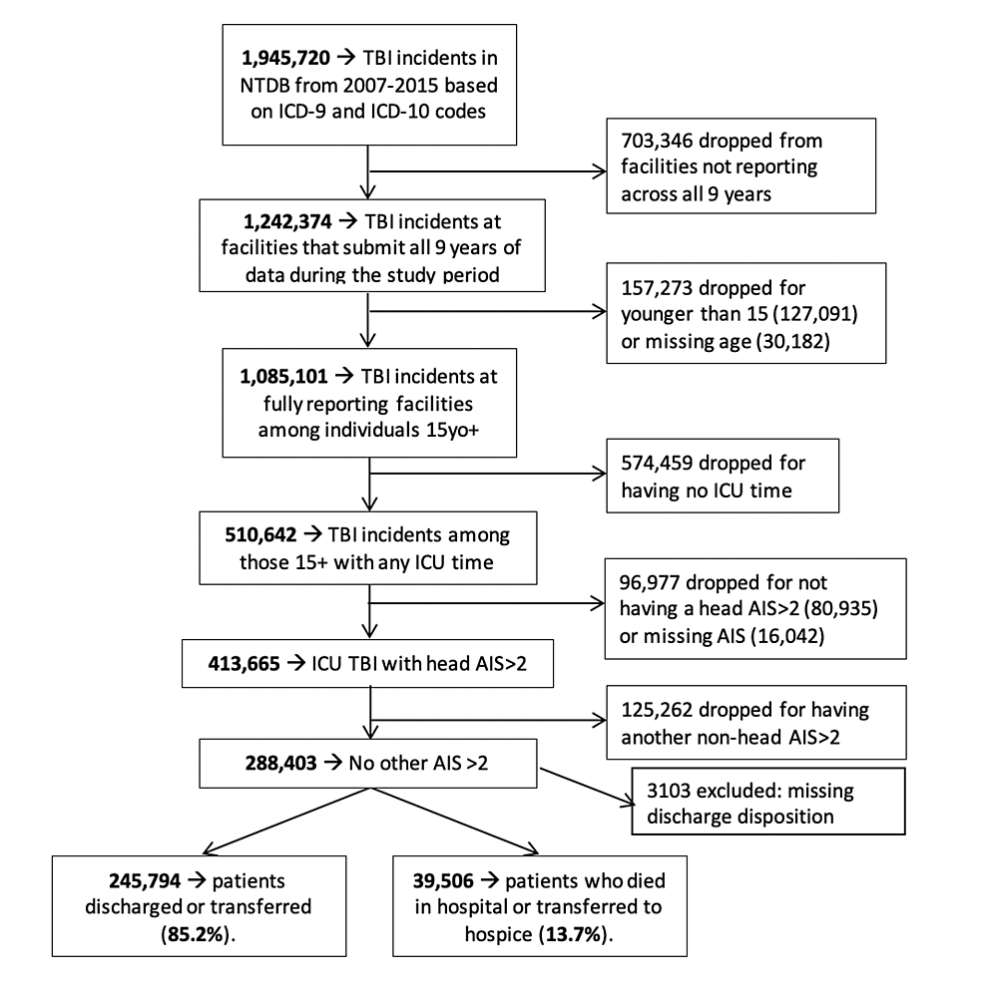
Moderate-severe traumatic brain injury patient selection from the National Trauma Data Bank (NTDB) for dataset creation (2007-2015) TBI: traumatic brain injury; ICD: International Classification of Diseases; ICU: Intensive Care Unit; AIS: Abbreviated Injury Scale

As shown in Figure 1, there were 1,945,720 TBI incidents in NTDB from 2007-2015 by ICD.9/ICD.10 codes. After excluding data from facilities not reporting data for all nine years, patients with age < 15 years or missing age, no ICU admission, non-head AIS>2 or another body region AIS>2, and those with missing discharge disposition, the final sample consisted of 285,300 patients (Table 3) between 15 and 85+ years. 

**Table 3:** Characteristics of patients with moderate-severe traumatic brain injury reported to the National Trauma Data Bank between 2007-2015 IQR: interquartile range [25th, 75th quartile]; ICU: intensive care unit

	Cohort N = 285,300 n (%); Median [IQR]
Male sex	192,597 (67.5)
Female sex	92,703(32.5)
Age	
15-24 years old	39,116 (13.7)
25-34 years old	30,748 (10.8)
35-44 years old	27,190 ( 9.5)
45-54 years old	37,983 (13.3)
55-64 years old	38,938 (13.6)
65-74 years old	38,115 (13.4)
75-84 years old	49,581 (17.4)
85+ years old	23,629 ( 8.3)
Transferred	113,603 (39.8)
Poly-Trauma	171,000 (59.9)
Hospital Length of Stay	8.3 [3, 10]
ICU Length of Stay	4.4 [1, 5]
Good Discharge Outcome	169,353 (59.4)
Died or Discharged to Hospice	39,506 (13.8)

NIH funding for clinical TBI research 2007-2015

During the study period, annual NIH funding for TBI varied from the lowest level of $48.79 million (in 2007) to the highest level of $77.34.43 million (in 2005), and funding estimates adjusted using the gross domestic product (GDP)-based annual implicit price deflation from the year 2000 (Table 4). The mean yearly funding during the study period was $67.06 million. 

**Table 4:** Moderate-severe traumatic brain injury-related incident mortality in the National Trauma Data Bank by Year, 2007-2015 ^Funding estimates adjusted using GDP-based annual implicit price deflation USD: United States dollars, GDP: gross domestic product

Year	Total Incidents	Died or Discharged to Hospice (n)	Mortality (%)	Research funding in contemporaneous USD	Research funding in 2000 USD^
2007	24,945	3,529	14.15	$82,446,000	$69,600,486
2008	27,410	3,812	13.91	$58,929,131	$48,794,673
2009	30,516	4,225	13.85	$83,395,377	$68,534,314
2010	32,746	4,555	13.91	$93,192,113	$75,705,517
2011	32,822	4,472	13.63	$80,996,575	$64,451,409
2012	33,738	4,810	14.26	$78,122,277	$60,993,364
2013	33,703	4,586	13.61	$69,084,361	$53,008,101
2014	35,495	4,983	14.04	$88,602,901	$66,723,735
2015	33,925	4,534	13.36	$95,881,204	$71,437,845

TBI-related mortality

**Figure 2:**
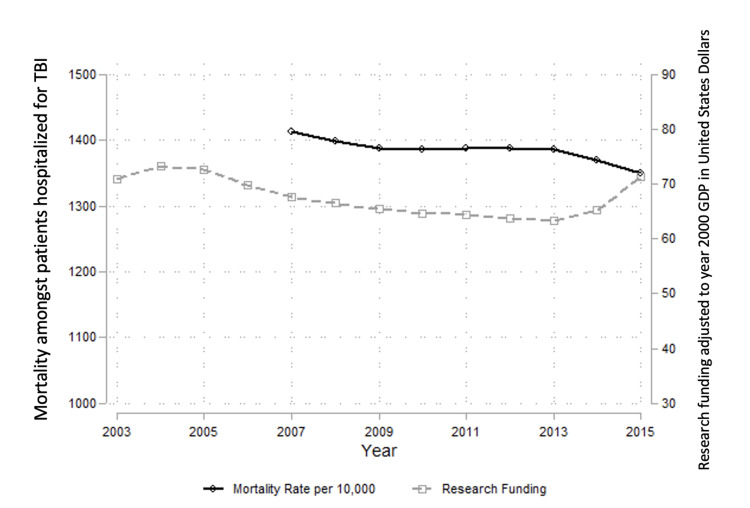
Trends in TBI-related all-cause in-hospital mortality and clinical research funding for traumatic brain injury in the United States, 2007-2015 TBI: traumatic brain injury, GDP: Gross Domestic Product

TBI-related incident mortality (Table 4) was variable; highest (1,426 deaths per 10,000 hospitalizations) in 2012 to lowest (1,336 deaths per 10,000 hospitalizations) in 2015 with no statistically significant linear trend from 2007 to 2015 (an average of 4.77 fewer deaths per 10,000 hospitalizations per year, 95% CI: -12.97, 3.42, Figure 2). 

**Figure 3:**
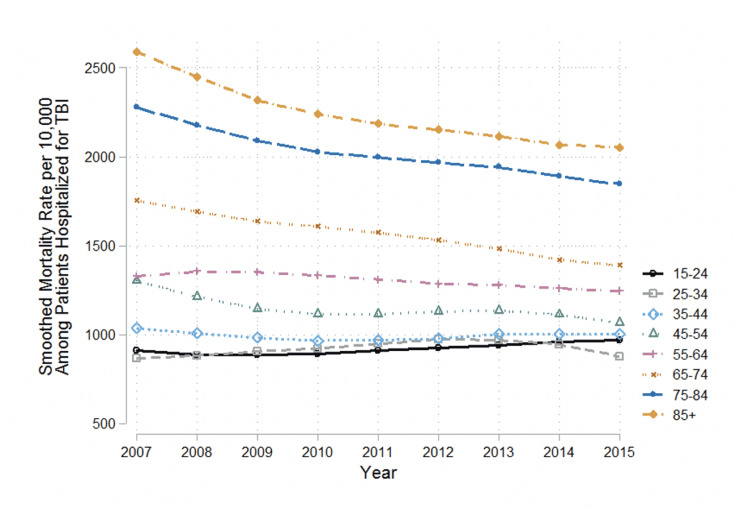
The TBI-related all-cause in-hospital mortality rate among hospitalized, isolated TBI patients (15-85+) in the NTDB, 2007-2015 by age group NTDB: National Trauma Data Bank

When stratified by age groups, there were statistically significant reductions in the estimated mean change in death rate per 10,000 TBI hospitalizations amongst those older than 55 years of age (Figure 3). Specifically, the greatest reduction was observed in those 85 years and older with an average reduction of 62.35 deaths per 10,000 hospitalizations per year over the study period (95% CI -92.45, -32.25), followed by patients 75-84 years (-44.41, 95% CI -61.72, -27.09), 65-74 years (-47.60, 95% CI -67.39, -27.81), and 55-64 years of age (-15.15, 95% CI -27.59, -2.72, Table 5).

**Table 5:** TBI-related all-cause in-hospital mortality in the National Trauma Data Bank by year, 2007-2015 ^Funding estimates adjusted using GDP-based annual implicit price deflation USD: United States dollars, GDP: Gross Domestic Product; TBI: traumatic brain injury

Year	Total Incidents	Died or Discharged to Hospice (n)	Mortality Rate per 10,000	Research funding in contemporaneous USD	Research funding in 2000 USD^
2004	--	--	--	$80,424,000	$74,067,962
2005	--	--	--	$86,591,000	$77,337,526
2006	--	--	--	$85,372,000	$74,006,131
2007	24,945	3,529	1,415	$82,446,000	$69,600,486
2008	27,410	3,812	1,391	$58,929,131	$48,794,673
2009	30,516	4,225	1,385	$83,395,377	$68,534,314
2010	32,746	4,555	1,391	$93,192,113	$75,705,517
2011	32,822	4,472	1,363	$80,996,575	$64,451,409
2012	33,738	4,810	1,426	$78,122,277	$60,993,364
2013	33,703	4,586	1,361	$69,084,361	$53,008,101
2014	35,495	4,983	1,404	$88,602,901	$66,723,735
2015	33,925	4,534	1,336	$95,881,204	$71,437,845

Association between NIH funding and in-hospital TBI-related mortality

There was no evidence of a statistically significant association between in-hospital severe-TBI-related deaths and NIH clinical research funding levels for TBI for the prior three years overall (p=0.52) or by ten-year age groups (Table 6). 

**Table 6:** Trends in TBI-related all-cause in-hospital mortality TBI-related all-cause in-hospital mortality (15-85+) in the National Trauma Data Bank, between 2007-2015 by age group TBI: traumatic brain injury

Age group	Estimated mean change in death rate per 10,000 TBI hospitalizations per year	(95% confidence interval)
All (15-85+)	-4.77	(-12.97, 3.42)
15-24	11.39	(-1.52, 24.29)
25-34	9.60	(-9.72, 28.91)
35-44	-0.68	(-23.14, 21.79)
45-54	-17.39	(-36.97, 2.18)
55-64	-15.15	(-27.59, -2.72)
65-74	-44.41	(-61.72, -27.09)
75-84	-47.60	(-67.39, -27.81)
85+	-62.35	(-92.45, -32.25)

## Discussion

In this study, we examined trends in and the relationship between NIH funding for clinical TBI research three years prior and all-cause in-hospital TBI mortality in the U.S between 2007-2015. The main findings are 1) NIH funding for clinical TBI research fluctuated over the eight-year study period, 2) overall all-cause in-hospital TBI mortality did not decrease, and 3) when stratified by age group, in-hospital mortality decreased only amongst patients older than 55 years; most pronounced in the >85-year age group. There was no statistical indication of an overall relationship between all-cause in-hospital TBI mortality and funding in the previous three years, indicating the need for efforts beyond NIH funding to achieve timely improvements for all TBI patients.

The study finds a reduction in all-cause in-hospital TBI mortality between 2007-2015 only for patients >55 years of age. Upstream, this variability may be due to known differences in mechanism, physiology and pathophysiology [1], which result in variable hospital trajectories. For example, older patients sustaining TBI after a fall may have comparatively more benefit from hospitalization than younger patients who sustain severe injuries from a motor vehicle crash and have an a priori higher risk of death. In-hospital variability in outcomes such as mortality between the age groups may reflect variability in treatments of TBI, which may be comparatively more or less evidence-based or advanced. Alternately, hospital treatments may or may not alter mortality risk at presentation. Our data sources did not allow us to examine TBI progression in relation to the mechanism of injury to offset any improvement from in-hospital care or research activities that may benefit patients. Despite the higher risk of death among older adults, as shown by Krishnamoorthy et al. [12], results of this study show that TBI-related mortality for hospitalized patients has decreased for the elderly over time. The lack of a strong association with funding in the prior three years suggests that clinical benefit from increased funding might operate on a longer timescale, and our findings also suggest that greater attention should be paid to ensuring quick translation of research into practice [13]. 

Our methodology in extracting NIH funding merits discussion. Examining the NIH RePORTER database, we used search terms that solely indicated clinical research funding and not pre-clinical research because it is more likely that the conduct of clinical or patient-oriented TBI research would more efficiently translate to changes in TBI care that improves TBI outcomes. Associating TBI NIH funding allocation with patient-level outcomes is appealing because it reflects a need to evaluate the impact of peer-reviewed research. However, these are notable limitations. First is the issue of NIH’s allocation pool not being mutually exclusive to TBI research only in that a research project may overlap two or more disease categories and disease severity (mild vs. moderate vs. moderate-severe) and hence may misrepresent its weight in terms of funding allocation. Second, NIH’s Research, Condition, and Disease Categories (RCDC) stratifies research funding allocation according to disease burden. In this case, funding for “all accidental injuries” from 2007 to 2015 shows a linear, year-on-year increase ($229 million in 2007 to $449 million in 2016 - an almost 100% increase) [7], a clear reflection of the public health priority for overall traumatic injuries. However, this pattern is not replicated for the subcategory of TBI, with one of the highest case fatality rates within the traumatic injuries group. 

The lack of observed statistical association between funding and improvements in mortality observed at the ecological level does not mean that more granular benefits at the faculty level have not occurred. For example, a large-scale review of the UK’s National Institute for Health Research (NIHR) and Comprehensive Clinical Research Network (CCRN) reviewed an annual allocation of £285M and 2.3 million hospital admissions. They showed that institutions with higher research funding had lower mortality outcomes for emergency admissions than institutions with lower research funding [14]. Study observations were that the risk-adjusted odds of lower mortality could not be explained by either staffing, critical care, operating theatre provision or neuro-imaging diagnostic utilization alone and that there was likely no one dominant responsible factor. In the United States, an analysis of primary care practice-based research networks (PBRNs) showed improvements in clinical outcomes in the participating practices versus non-PBRN institutions [15]. This positive change in outcomes was attributed to PBRN’s being an effective vehicle for education, translation, and practice change, in addition to their value in research. Similarly, a German study showed that institutions conducting clinical trials demonstrated improved survival even in subjects not included in the studies [16]. They hypothesized that incorporating research in any organization leads to the development of special infrastructure, resulting in overall improvement of care. Additionally, teaching institutions with a larger share of research may be better and faster in adopting novel treatments when compared to their counterparts with lesser research activity [17]. While maybe, institutions that receive NIH funding for clinical TBI research are often teaching and trauma centers, greater benefits for TBI patients may be observed when TBI care is provided in these facility types. There are likely mediators between funding availability and outcomes that need to be further examined to understand this relationship better.

There are some additional study limitations and strengths beyond the important point that our study is ecological. Limitations are inherent limitations of the data sources; the NIH RePORTER database is drawn from several databases but does not encompass all federally funded research; hence we may have missed potential funding sources. There is also the possibility of under or over-reporting of funds towards TBI as NIH RePORTER allocations are not mutually exclusive between multiple projects. Regarding the NTDB database, weakness pertains to it being an administrative, subjective, self-reporting database, not primarily for research leading to inconsistent reporting, human error, and other biases. The NTDB represents a national sample of hospitals that choose to report their data to the NTDB, and thus the data are not nationally representative, and it is possible that centers that do not choose to report to the NTDB may experience different results than observed in this study. We only included data from trauma centers that reported to NTDB in all nine years were included in the study, which further limited the sample size. We also cannot comment on increased proficiency in TBI care of the elderly, which may influence the reduction in mortality numbers. We could not distill research relevant to more upstream TBI care than the hospital setting, such as the emergency department or clinic, but assumed that improvements in preadmission health care settings could improve hospital survival. We could not attribute research location or research topic to our observation of associated improved hospital discharge survival for patients hospitalized with TBI but assumed that all patients hospitalized with TBI are at risk of deterioration across hospital settings. The main strength is the novel use of publicly available data, both NIH RePORTER and NTDB, to understand the impact of research on clinical outcomes in TBI. These data also likely reflect death related to TBI and not polytrauma as we excluded patients with AIS scores greater than 2 in body regions other than the head. These findings add to our understanding of elderly patient outcomes in TBI over a recent eight-year period. 

## Conclusions

This study finds a variable pattern in NIH funding for clinical TBI research and a contemporaneous reduction in moderate-severe TBI-related deaths for those only aged 55 years and older, but no association between in-hospital TBI-related mortality rate and funding in the prior three years. This study is a first attempt to understand whether national-level clinical research funding translates to timely patient benefit in TBI.
