# Correction: National Institutes of Health Clinical Research Funding and All-Cause In-Hospital Traumatic Brain Injury-Related Mortality

**DOI:** 10.7759/cureus.c77

**Published:** 2022-10-05

**Authors:** Anwar Alinani, Brianna Mills, Emma Gause, Monica S Vavilala, Abhijit V Lele

**Affiliations:** 1 Anesthesiology and Pain Medicine, Harborview Medical Center, Seattle, USA; 2 Anesthesiology and Pain Medicine, Harborview Injury Prevention and Research Center, Seattle, USA; 3 Anesthesiology and Pain Medicine, Harborview Injury Prevcention and Research Center, Seattle, USA; 4 Neurocritical Care/Anesthesiology, Harborview Medical Center, Seattle, USA

This article has been corrected at the request of the authors to correct a typo in the y-axis label of Figure 2. The authors and the journal regret that this typo was not recognized and corrected prior to publication.

